# 

**Published:** 1897-05-08

**Authors:** 


					uiii, mmi iial.
r\ A HOI I ^UKe| therefore BEST. May 8th, 1897.
/ CAW.? It* OADcf^RY's,
1 The standard of highest purity at present
attainable."?Lancet.
No. 55 j Yol. XXII. [faffper!] Saturday,
May 8th, 1897.
SttbscBeep?90 S,J PRICE TWOPENCE.

				

